# CTGC motifs within the HIV core promoter specify Tat-responsive pre-initiation complexes

**DOI:** 10.1186/1742-4690-9-62

**Published:** 2012-07-26

**Authors:** Emmanuelle Wilhelm, Marie-Christine Doyle, Isaac Nzaramba, Alexandre Magdzinski, Nancy Dumais, Brendan Bell

**Affiliations:** 1 RNA Group. Département de microbiologie et d’infectiologie, Faculté de médecine et sciences de la santé, Université de Sherbrooke, Pavillon de recherche appliquée sur le cancer, 3201 rue Jean-Migneault, Sherbrooke, Québec, J1E 4K8, Canada; 2 Département de biologie, Faculté des sciences, Université de Sherbrooke, 2500, boulevard de l'Université, Sherbrooke, Québec, J1K 2R1, Canada

## Abstract

**Background:**

HIV latency is an obstacle for the eradication of HIV from infected individuals. Stable post-integration latency is controlled principally at the level of transcription. The HIV trans-activating protein, Tat, plays a key function in enhancing HIV transcriptional elongation. The HIV core promoter is specifically required for Tat-mediated *trans*-activation of HIV transcription. In addition, the HIV core promoter has been shown to be a potential anti-HIV drug target. Despite the pivotal role of the HIV core promoter in the control of HIV gene expression, the molecular mechanisms that couple Tat function specifically to the HIV core promoter remain unknown.

**Results:**

Using electrophoretic mobility shift assays (EMSAs), the TATA box and adjacent sequences of HIV essential for Tat *trans*-activation were shown to form specific complexes with nuclear extracts from peripheral blood mononuclear cells, as well as from HeLa cells. These complexes, termed pre-initiation complexes of HIV (PICH), were distinct in composition and DNA binding specificity from those of prototypical eukaryotic TATA box regions such as Adenovirus major late promoter (AdMLP) or the hsp70 promoter. PICH contained basal transcription factors including TATA-binding protein and TFIIA. A mutational analysis revealed that CTGC motifs flanking the HIV TATA box are required for Tat *trans*-activation in living cells and correct PICH formation *in vitro*. The binding of known core promoter binding proteins AP-4 and USF-1 was found to be dispensable for Tat function. TAR RNA prevented stable binding of PICH-2, a complex that contains the general transcription factor TFIIA, to the HIV core promoter. The impact of TAR on PICH-2 specifically required its bulge sequence that is also known to interact with Tat.

**Conclusion:**

Our data reveal that CTGC DNA motifs flanking the HIV TATA box are required for correct formation of specific pre-initiation complexes *in vitro* and that these motifs are also required for Tat *trans*-activation in living cells. The impact of TAR RNA on PICH-2 stability provides a mechanistic link by which pre-initiation complex dynamics could be coupled to the formation of the nascent transcript by the elongating transcription complex. Together, these findings shed new light on the mechanisms by which the HIV core promoter specifically responds to Tat to activate HIV gene expression.

## Background

Latency of HIV contributes to viral persistence despite current antiviral therapies, as well as to immune evasion [1-4]. Post-integration latency is the most long-lived source of latent HIV where the host cell genome harbors a functional provirus that is transcriptionally silent [4,5]. The HIV promoter region, located within the 5’ long terminal repeat (LTR) of the integrated viral genome, pirates the host cell RNA polymerase II (Pol II) machinery to initiate viral transcription. Transcriptional interference, chromatin structure and modification (e.g. acetylation/deacetylation of histones), DNA methylation, limitation of host factors (e.g. P-TEFb), and activation by specific host cell factors (e.g. NF-κB, NFAT1) can all contribute to the activation of HIV transcription [1,3,4]. The core promoter is ultimately the gateway for all signals activating HIV transcription, since it nucleates the formation of Pol II pre-initiation complex (PIC) which is a rate-limiting step in the initiation of transcription [6].

Once HIV transcription occurs, the HIV Tat gene product can be expressed and plays an important role in the exit from latency by driving a feed-forward loop to fully activate viral transcription [7]. Tat acts by physically interacting with a stem-loop structure of the nascent 5’ HIV RNA termed TAR. Tat binds to TAR in conjunction with an essential cellular cofactor termed positive transcription elongation factor b (P-TEFb), composed of CDK9 and Cyclin T1 [8-10]. Recently published data suggest that nascent TAR RNA can displace promoter-bound transcription complexes containing an inhibitory small non-coding RNA (7SK) that sequesters P-TEFb in an inactive form [11]. Once in the active, 7SK free form, P-TEFb is recruited by Tat into a complex with other cellular elongation factors and co-factors termed the super elongation complex (SEC) [12,13]. Tat acts within SEC on Pol II, in part via phosphorylation of the c-terminal domain (CTD) of Pol II, to potently increase elongation rates of HIV transcription [14,15]. The HIV *trans*-activator Tat thus drives an amplification loop during the reactivation of latent HIV.

**Figure 1:**
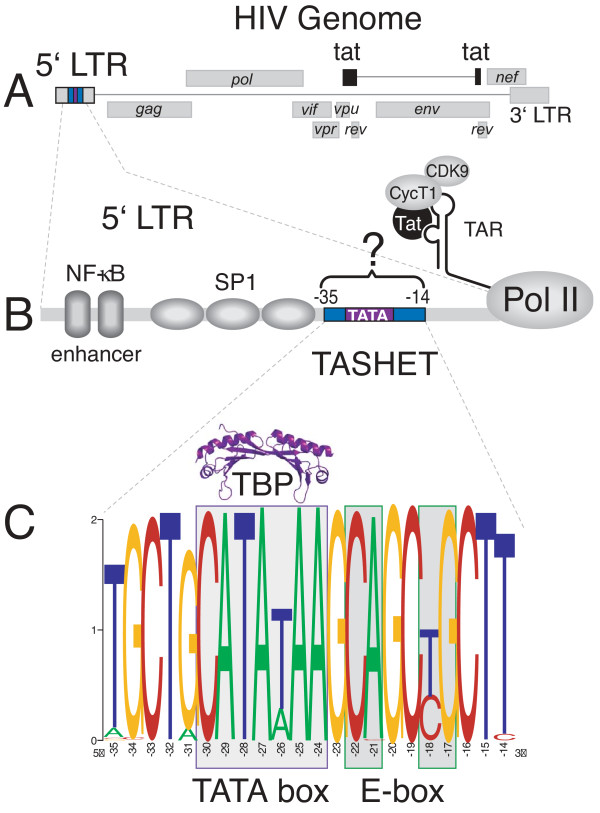
**Natural sequence variation of the HIV core promoter. (A)** The HIV genome is shown in schematized form. The gene encoding the viral trans-activating protein, Tat, is in black. The core promoter of the 5’ long terminal repeat (LTR) is in blue. **(B)** A schematized version of the HIV promoter (5’ LTR) is shown with cellular transcription factors in grey. Viral Tat protein is shown in black interacting with the TAR stem-loop RNA structure at the 5’ end of the nascent HIV transcript. The TATA box and adjacent sequences of HIV essential for Tat *trans*-activation (TASHET) contain the TATA box (purple) and flanking sequences (blue). A question mark highlights a mechanistic missing link in our current understanding of the specific requirement for TASHET in Tat *trans*-activation. **(C)** Natural sequence variation of TASHET (positions – 35 to – 14 relative to the transcription start site) is shown. HIV sequences from the Los Alamos HIV Sequence Database (http://www.hiv.lanl.gov) were aligned, and the weblogo algorithm (http://weblogo.berkeley.edu/) was used to generate a logo sequence in which the height of each nucleotide position indicates sequence conservation. The height of different nucleotides at single position indicates their relative frequency.

In addition to the essential role of TAR RNA in Tat activity, a DNA element within the HIV core promoter has been shown to be specifically required for Tat *trans*-activation [16-20]. Berkhout and Jeang published the first demonstration that replacement of the HIV TATA box region with other eukaryotic TATA box regions strongly reduced Tat-activation with no pronounced effects on basal transcription or transcriptional start site selection [16]. Likewise, Olsen and Rosen found that minor nucleotide changes within or immediately flanking the TATA motif diminished Tat-mediated *trans*-activation without affecting transcription initiation site or transcriptional activation via the upstream NF-κB sites [19]. Lu *et al.* showed that the HIV TATA box and immediately flanking sequences are essential for the production of non-processive transcripts that are targets of *trans*-activation by Tat [18]. Montanuy *et al*. confirmed and extended these findings, showing that the HIV TATA box is required to enhance transcriptional elongation in response to recruitment of the P-TEFb subunit, CDK9 [21]. Even in the context of a heterologous thymidine kinase herpes simplex promoter where Tat is recruited via DNA elements, the HIV TATA box region was required for optimal Tat *trans*-activation [17]. Sequences flanking the HIV TATA box, particularly those overlapping a 3’ E-box that can bind cellular transcription factor AP4, have been shown to be critical for Tat-*trans*-activation [20]. More recently AP4, a bHLH domain containing transcriptional factor, was shown not to be required for Tat *trans*-activation, but instead to repress HIV transcription in part by competing with TATA-binding protein (TBP) for the HIV core promoter [22]. Recruitment of TBP to the HIV LTR is necessary, but not sufficient, for Tat *trans*-activation [23,24]. Molecular genetic studies indicate that TBP recognizes a CATA sequence rather than a canonical TATA sequence within the HIV core promoter [25]. HIV Tat can physically interact with TBP [26-28], although the requirement of sequences surrounding the HIV TATA box in Tat *trans*-activation suggests that cellular factors other than TBP must recognize the core promoter in order for Tat to act upon it. Importantly, a proof-of-principle that the HIV core promoter represents a therapeutically useful target has been provided using polyamides that bind specifically to GC(T/A)GC motifs flanking the HIV TATA box [29]. These molecules repressed HIV replication in primary lymphocytes without any apparent reduction in cell viability [29]. Taken together, the above studies demonstrate that the unique architecture of the HIV TATA box and flanking sequences are essential for Tat *trans*-activation. To distinguish Tat-responsive sequences from the entire HIV core promoter, and for brevity, we refer hereafter to these sequences as the TATA box and adjacent sequences of HIV essential for Tat *trans*-activation (TASHET) (Figure 1).

TASHET plays a crucial role in the control of HIV gene expression and latency; yet, the cellular complexes that impart functional specificity upon it are completely unknown. The identification of specific cellular TASHET-binding complexes could potentially reveal drug targets for therapies to flush out, or definitively silence, latent virus in HIV infected individuals. Given the deepening realization that core promoter binding complexes are highly diverse [6,30-35], together with recent advances in the experimental detection of endogenous pre-initiation complexes [36], we have revisited TASHET function. Here, we report the results of experiments designed to answer three critical but unresolved questions: 1) Are cellular pre-initiation complexes that recognize TASHET distinct from those binding the other eukaryotic TATA elements such as the prototypical Adenovirus major late promoter (AdMLP)? 2) Precisely what *cis*-acting DNA sequences are required for the formation of Tat-responsive pre-initiation complexes? 3) What physical interactions account for the functional dependence of the Tat/TAR axis on TASHET?

## Results

### Natural sequence variation of the TASHET element of the HIV core promoter

The availability of extensive sequencing of HIV promoters from clinical isolates provides a wealth of information about the conservation of nucleotides that are important for viral replication. To establish the sequence variation within the HIV core promoter (Figure 1A and 1B), we aligned HIV promoter sequences from the Los Alamos HIV Sequence Database (http://www.hiv.lanl.gov). We focused on nucleotide conservation corresponding to the TATA box and adjacent sequences of HIV essential for Tat *trans*-activation (TASHET). Nucleotides −33 to −16 with respect to the transcriptional start site displayed mostly high levels of conservation while nucleotides flanking this region displayed decreasing conservation. The conservation of most nucleotides from −33 to −16 corresponds well with the core promoter sequences defined by several independent mutational studies to be essential for Tat *trans*-activation [16-21,37]. Two nucleotides within TASHET that display notably high variability include the previously noted position −26 (T or A) within the TATA box [38,39] and position −18 (T or C) within an E-box element immediately downstream (Figure 1C). The conservation of TASHET sequences from naturally occurring HIV isolates guided our design of mutations to identify functionally important base-pairs throughout this study.

### Host cell complexes formed on TASHET are distinct from those forming on the AdMLP

**Figure 2:**
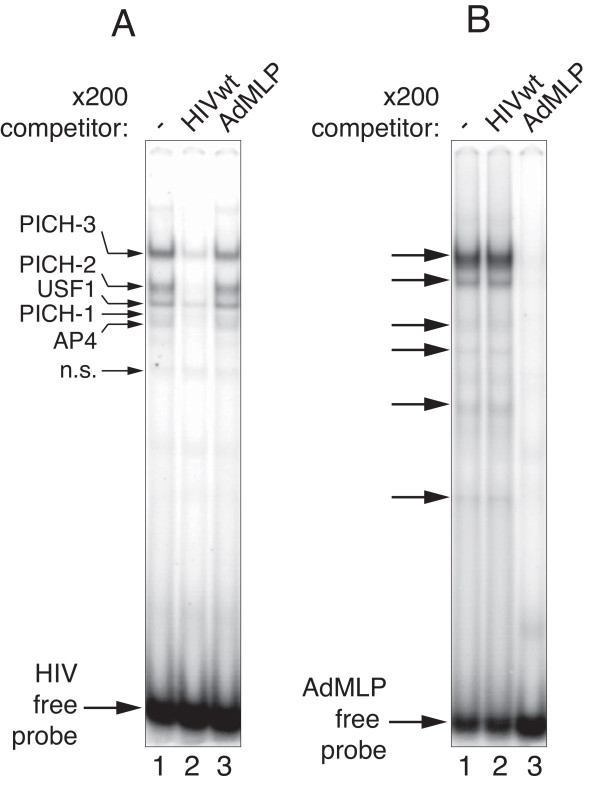
**TASHET of the HIV core promoter binds nuclear complexes distinct from those binding the canonical Adenovirus major late promoter (AdMLP). (A)** Radiolabelled oligonucleotides corresponding to the HIV core promoter were incubated with HeLa nuclear extracts and the resulting complexes were separated on a native polyacrylamide gel. Bound complexes were analyzed by phosphorimaging. The binding reactions were challenged by the addition of a 200 fold molar excess of unlabelled HIV wt promoter (lane 2), or AdMLP (lane 3). Pre-initiation complexes of HIV (PICH) and other HIV core promoter-binding cellular factors are labelled at the left. n.s. indicates a non-specific complex. The free probe is shown with an arrow at the bottom of the gel. **(B)** As in A except that radiolabelled AdMLP was the probe. Specific complexes are indicated with arrows in both panels.

We have recently reported electrophoretic mobility shift assay (EMSA) conditions that are optimised to allow the detection of endogenous RNA Pol II pre-initiation complexes (PIC) [36]. To test experimentally whether transcription complexes formed on the TASHET are distinct from those that form on the model AdMLP TATA region, EMSAs were performed under conditions that detect endogenous PIC with radiolabelled double-stranded TASHET and equivalent oligonucleotides from the AdMLP TATA region to probe the nuclear complexes that recognize them. Radiolabelled TASHET formed complexes with nuclear extracts from HeLa cells (Figure 2A, lane 1). We applied a nomenclature for the distinct TASHET-binding complexes based on the identification of their subunits as described below. Competition with a two hundred fold molar excess of unlabelled TASHET prevented the formation of the majority of the observed complexes, showing their specificity (Figure 2A, lane 2). In stark contrast, addition of an identical excess of unlabelled AdMLP oligonucleotide had no significant impact on the formation of the same complexes (Figure 2A, lane 3). Conversely, radiolabelled AdMLP formed complexes with HeLa cell nuclear factors that were distinct from those binding the TASHET in their electrophoretic mobility (Figure 2B, lane 1). These complexes were unaffected by the addition of a two hundred fold molar excess of unlabelled TASHET DNA (Figure 2B, lane 2), but were effectively blocked by the addition of unlabelled AdMLP (Figure 2B, lane 3). We concluded that cellular complexes distinct in composition and in DNA binding specificity from those that recognize the equivalent TATA box region from the prototype AdMLP recognize the TASHET.

### Pre-initiation complexes of HIV (PICH) from HeLa cells and from PBMCs specifically recognize TASHET *in vitro*

**Figure 3:**
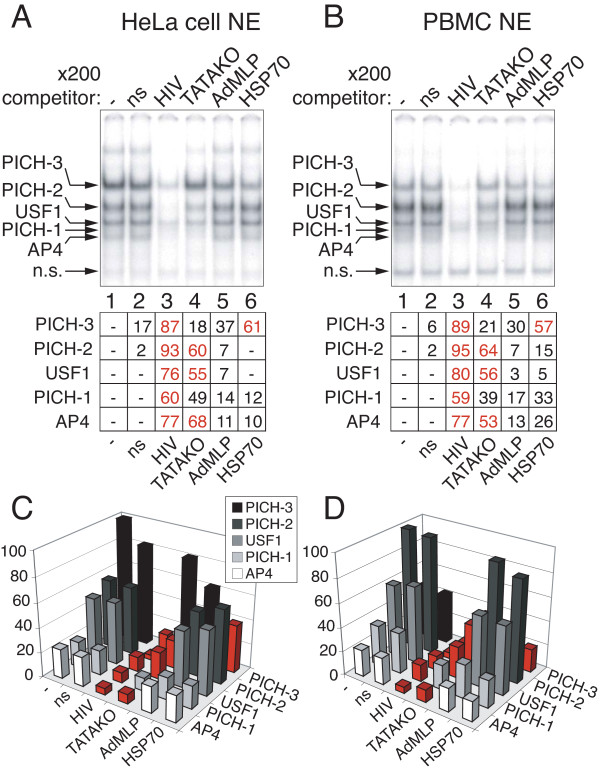
**Pre-initiation complexes of HIV (PICH) from PBMC and HeLa cells specifically recognize the intact HIV wt core promoter.** EMSAs were performed as in Figure 2. **(A)** HeLa nuclear extracts were used in binding reactions that were challenged by the addition of a 200 fold molar excess of the indicated unlabelled promoter competitors (lane 2 to 6). Lane 1 contains no competitor (−). Lane 2 contains a scrambled non specific competitor. The upper panel shows complexes in the EMSA reaction. Pre-initiation complexes of HIV (PICHs) and a non-specific complex are indicated by arrows at the left. Quantifications of EMSA bands are displayed in the table in the lower panel, expressed as the percentage of reduction of each complex’s intensity upon competition. Complexes whose intensities are reduced by 50% or more by the addition of unlabelled competing oligonucleotides are highlighted in red. **(B)** As in A except that nuclear extracts from activated PBMC were used. **(C)** Quantification of EMSA band intensities from panel A is shown in graphic form. The intensity of the most abundant PICH in the control lane (panel A lane 1) has been set to 100%. Red color is used to highlight reduction of 50% or more for each complex relative to its level in the control lane (−). Shades of grey are used to distinguish each PICH. Relative band intensity is given on the vertical Y-axis, complexes are indicated on the right (Z-axis), and competitor oligonucleotides on the left (X-axis). **(D)** As in C, except that the graph represents the relative band intensities of the EMSAs from panel B.

To further investigate the cellular complexes that specifically recognize the TASHET element within the HIV core promoter, we performed competition with the equivalent TATA box region from the cellular hsp70 promoter. As with the AdMLP, (Figure 3, lane 5), the hsp70 promoter was unable to compete for all complexes binding the TASHET with the exception of PICH-3 that showed a modest competition (Figure 3, lane 6). The fact that complexes recognizing the TASHET are distinct in DNA binding specificity from those that recognize the cellular hsp70 TATA box region lends further support for the atypical nature of these complexes. Given the unusual nature of the TASHET-binding complexes, we next tested whether they require the TATA box itself for binding. Unlabelled competitor DNA lacking a TATA box competed very poorly for binding to PICH-3, and competed less effectively for other complexes (Figure 3, lane 4 “TATAKO”) than the wild type TASHET (Figure 3, lane 3). We concluded that PICH-3 formation requires an intact TATA box to bind to the HIV core promoter.

The HeLa cell model has been employed as a valuable model to identify cellular transcription factors that control HIV transcription such as SP1 [40], NF-κB [41], and P-TEFb [9]. To examine the suitability of the HeLa cell system for the study of cellular complexes binding to TASHET, we isolated nuclear extracts from activated peripheral blood mononuclear cells (PBMC) from healthy donors as these contain the physiological targets for HIV infection CD4+ lymphocytes and monocytes. EMSA assays were performed with PBMC nuclear extracts and revealed complexes of indistinguishable mobility and DNA binding specificity compared to those from HeLa cell nuclear extracts (Figure 3, panel B, lanes 1–6 versus panel A lanes 1–6). The similarities between TASHET-binding complexes from HeLa cells and PBMC support the suitability of HeLa cells as a model system for the study of host cell TASHET-binding complexes.

**Figure 4:**
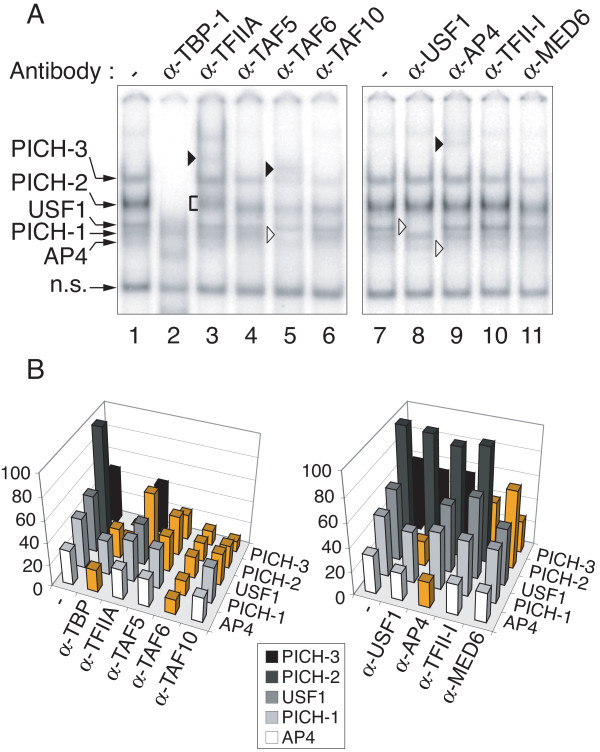
**PICH formation from PBMC nuclear extracts depends on general transcription factors.** EMSAs were performed with PBMC nuclear extracts as in Figure 2. **(A)** The indicated antibodies were added to the reaction (lanes 2 to 6 and 8 to 11). Lanes 1 and 7 contain no antibody. Pre-initiation Complexes of HIV (PICHs), other complexes and a non-specific complex are indicated by arrows at the left. Black arrowheads highlight supershifted bands. Brackets point out smearing of bands. White arrowheads highlight bands lost upon antibody addition. **(B)** Quantification of EMSA band intensities from panel A. Band intensities are expressed on the Y-axis as a percentage relative to the strongest band in the control lane (panel A lane 1 or 7) that was set at 100%. The graph on the left corresponds to lanes 1 to 6, and the graph on the right to lanes 7 to 11. Band intensities that change by one third (33%) or more relative to its level in the control lane (−) are highlighted in orange. Complexes are displayed on the right (Z-axis), supershifting antibodies on the left (X-axis). Shades of grey are used to distinguish each PICH.

To shed light on the identity of the cellular complexes that recognize the TASHET in a TATA box dependent fashion, we next performed a series of supershift experiments using nuclear extracts from PBMC. Monoclonal antibodies directed against human TATA-binding protein (TBP) caused a significant loss of binding of the majority of the TASHET-binding complexes (Figure 4A, lane 2 versus lane 1, see also Additional file 1 for quantification). We referred to the complexes that depend on one or more general transcription factors for their formation as PICH-1, PICH-2 and PICH-3, for pre-initiation complex of HIV one through three (Figure 4). The addition of TFIIA antibodies reproducibly caused a smearing and slower migration of PICH-2 (Figure 4, A, lane 3; see also Additional file 1, panel B), indicating that PICH-2 contains the basal Pol II transcription factor TFIIA. The addition of antibodies directed against TFIID subunits TAF5 and TAF10 to EMSA resulted in a reduction in the binding of PICH-2 and PICH-3 (Figure 4A, lanes 4 and 6 versus lane 1). Likewise, the addition of TAF6 antibodies caused a reduction in PICH-1, PICH-2 and PICH-3 binding and was accompanied by a supershifted complex (Figure 4A, lane 5 versus lane 1). We concluded that PICH-1, PICH-2 and PICH-3 are TFIID-dependent complexes. Antibodies directed against TFII-I had a modest negative effect on PICH-3 formation (Figure 4A, lane 10 versus lane 7), consistent with a recent report that TFII-I can interact with the HIV core promoter [42]. The addition of antibodies against the mediator subunit, MED6 also reduced binding of PICH-3 (Figure 4A, lane 11 versus lane 7), suggesting PICH-3 depends on the mediator complex for its stable association with TASHET. We also compared the effect of supershifts with TBP, TAF4, and TFIIA antibodies on TASHET with cellular pre-initiation complexes that bind the AdMLP. The results showed that all antibodies, especially anti-TFIIA, had a less dramatic effect on PIC bound to the AdMLP (Additional file 2). The above described supershift experiments show that the cellular PICH complexes that recognize TASHET contain classical Pol II basal transcription factors.

The presence of general Pol II transcription factors within PICH cannot by itself account for TASHET's specific capacity to respond to Tat [16-21,37]. Given reports that the E-box immediately downstream of the TATA box may be important in the response to Tat [20], we tested for the presence of two bHLH factors, AP4 [22] and USF-1 [42], that have been reported to bind to this E-box. Antibodies directed against AP4 resulted in the loss of a minor TASHET-interacting complex (Figure 4A, lane 9 versus lane 7 see also Additional file 1, panel B), in agreement with previous findings of AP4 interacting with the 3’ E-box [22]. The addition of USF-1 antibodies resulted in the loss of a complex that binds specifically to the TASHET (Figure 4A, lane 8 versus lane 7). Thus PBMC nuclear extracts contain the bHLH factors USF-1 and AP4 both of which are able to bind to TASHET in EMSA.

**Figure 5:**
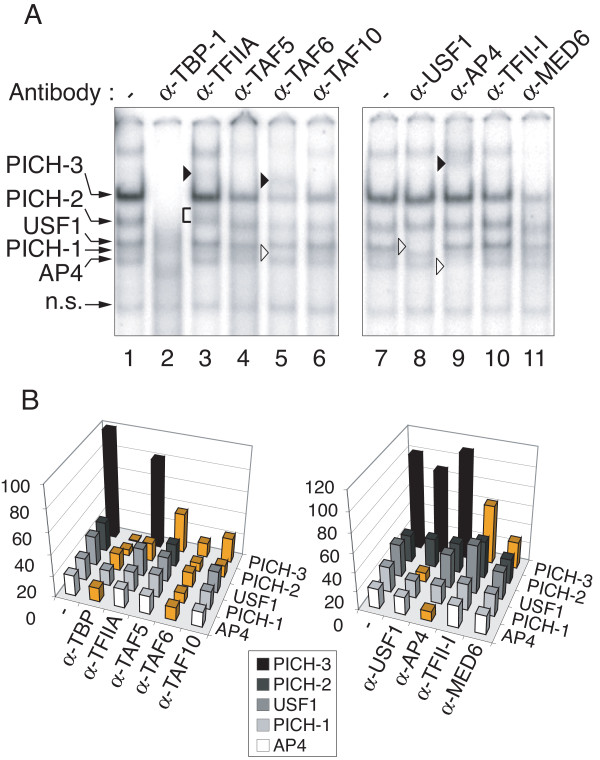
**PICH formation from HeLa nuclear extracts depends on general transcription factors.** As in Figure 4, except that EMSAs were performed with HeLa nuclear extracts.

We next tested whether PICH-1, -2, -3, USF-1 and AP4 from HeLa cell nuclear extracts also bound to TASHET using EMSA and supershift assays. As was the case of PBMC nuclear factors, antibodies against TBP reduced binding of all three PICH (Figure 5A, lane 2 versus lane 1). TFIIA antibodies also caused a similar change in PICH-2 mobility in HeLa and PBMC extracts (Figure 5 A, lane 3; see also Additional file 1, panel D). Indeed, in all cases supershift experiments with TFIID subunits, USF-1 and AP4 antibodies showed comparable effects on PBMC and HeLa nuclear complexes binding to TASHET (Figures 4 versus 5 and Additional file 1). The above results together reveal the existence of PICH from both HeLa cells and PBMC, distinct from canonical PIC, that specifically recognizes the TASHET.

### E-box binding factor USF-1 is dispensable for Tat *trans*-activation

**Figure 6:**
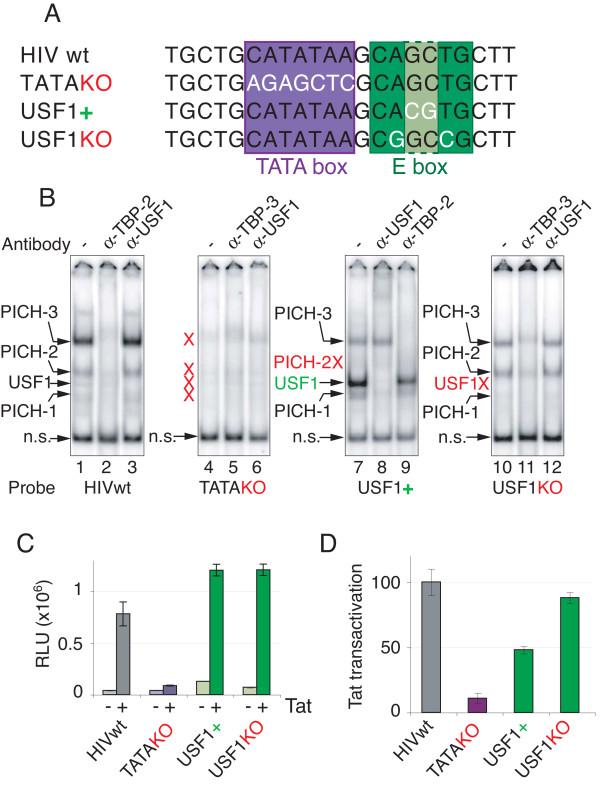
**USF-1 binds HIV core promoter but is dispensable for Tat*trans*-activation. (A)** Mutations in TASHET used in EMSA and transient transfections shown in B, C and D: the name of the mutation is indicated at the left of the sequence. The mutated nucleotides are indicated in white. The TATA box is delimitated by a purple box and the E-box by a green box: the lighter green region indicates nucleotides not forming part of the core E-box consensus sequence. **(B)** EMSAs were performed as in Figure 2. The different radiolabelled HIV promoter mutations used as a probe are indicated at the bottom of the figure. Supershift experiments were performed with the indicated antibodies. PICHs are indicated by an arrow at the left of each panel. An X in red indicates PICHs whose formation is severely impaired by the mutations. **(C)** HeLa cells have been co-transfected with a plasmid expressing the Renilla luciferase under the control of HIV wt or mutated promoter with or without a Tat expression plasmid as indicated under the X-axis. Luciferase activity was measured 48 h after transfection in cell extracts. Basal (−) and Tat-induced (+) activities are displayed. Mutation in the TATA box is shown in purple, mutations in the E-box are indicated in green. **(D)** As in C except that results are expressed as Tat *trans*-activation, obtained by the ratio of RLU in presence of Tat versus without Tat. Values are expressed as a percentage of wt HIV promoter luciferase activity (100%).

A previous mutational analysis suggested that the E-box immediately downstream of the HIV TATA box may be important for the response to Tat, potentially via binding of the bHLH factor AP4 [20]. A more recent study showed that AP4 is not required for Tat *trans*-activation [22]. To dissect in further depth the role of the 3’ E-box (Figure 6A) in the response to Tat, we designed point mutations predicted to either improve or abolish the binding of USF-1, since it represents the predominant E-box binding factor in nuclear extracts (Figures 4A and 5A lane 8 versus lane 7). Based on previous structure-function studies of USF-1 [43,44], we introduced a CA**CG**TG E-box (Figure 6A, “USF-1+”), with increased affinity for USF-1 with respect to the wild type HIV E-box, CAGCTG. BLAST searches showed that the CA**CG**TG E-box is extremely rare in natural HIV strains presumably because it is under negative selection pressure in HIV infected individuals. A second mutation was designed to prevent USF-1 binding but expected to retain replicative capacity based on sequence conservation (Figure 1C). Two nucleotide changes that exist individually in natural HIV isolates were introduced to produce a C**G**GC**C**G sequence (Figure 6A, “USFKO”) that is not expected to bind USF-1 [44]. These point mutations were tested for USF-1 binding with HeLa cell nuclear extracts. TASHET lacking a TATA box was used as a negative control and displayed virtually no PICH or USF-1 binding (Figure 6B, lanes 4–6). To confirm the identity of PICHs, TBP antibodies were used in supershift analysis as a positive control (Figure 6B, lanes 2, 5, 9 and 11). The CA**CG**TG (USF-1+) E-box bound more strongly to USF-1 (Figure 6B, lane 7 versus lane 1). The identity of USF-1 was confirmed by supershift analysis (Figure 6B, lane 8 versus lane 7). The C**G**GC**C**G (USFKO) sequence bound to PICH-3, PICH-2 and PICH-1 but, importantly, bound no USF-1 (Figure 6B, lane 10 versus lane 1). The increased affinity of the USF-1+ mutation for USF-1, as well as the lack of binding of the USFKO mutation to USF-1, was further reinforced using competition assays in EMSA (Additional file 3). To test the impact of these mutations on Tat *trans*-activation in living cells, we introduced them into HIV promoters driving *Renilla* luciferase as a reporter gene. As expected a construct lacking a functional TATA box did not respond to Tat co-transfection in HeLa cells (Figure 6C and 6D, TATAKO). The USF+ mutation decreased the Tat response by approximately two-fold, showing that increased USF-1 binding did not enhance Tat activation. Importantly, the USF-1KO mutation that did not bind USF-1 resulted in wild type levels of Tat *trans*-activation in living cells. These results show that USF-1 binding to TASHET is not required for Tat responsiveness.

### CTGC DNA motifs are required for correct PICH formation *in vitro* and Tat *trans*-activation in living cells

**Figure 7:**
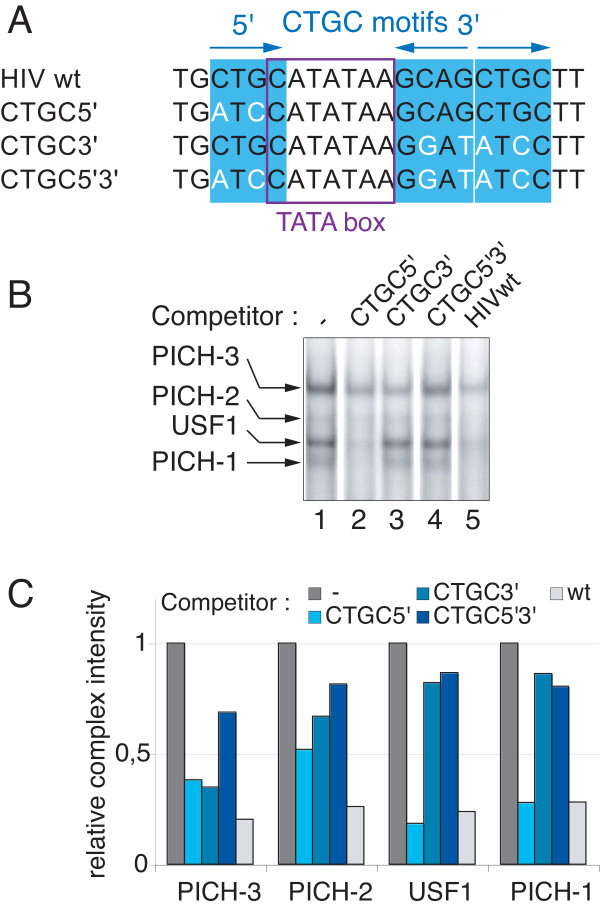
**CTGC DNA motifs are essential for the stable formation of PICH-1, -2 and −3 on TASHET*in vitro*.** EMSAs were performed as detailed in Figure 2. **(A)** Mutations used in EMSA and transient transfections shown in Figures 7 and 8: The name of the mutation is indicated at the left of the sequence. The mutated nucleotides are indicated in white. The purple box indicates the TATA box. The blue boxes indicate the CTGC motifs: the CTGC5’ motif is located just upstream of the TATA box, and two CTGC3’ motifs are located just downstream of the TATA box, the first one in the reverse orientation, the second one in the forward orientation. The mutations are named after the displayed mutations. **(B)** The indicated unlabelled competitors were added to the reaction (lanes 2 to 5). Lane 1 contains no competitor. **(C)** Relative intensity of the different complexes was calculated by phosphorimager analysis of the EMSA shown in B. Results are expressed relative to the intensity observed without competitor that was set to 1 for each complex.

**Figure 8:**
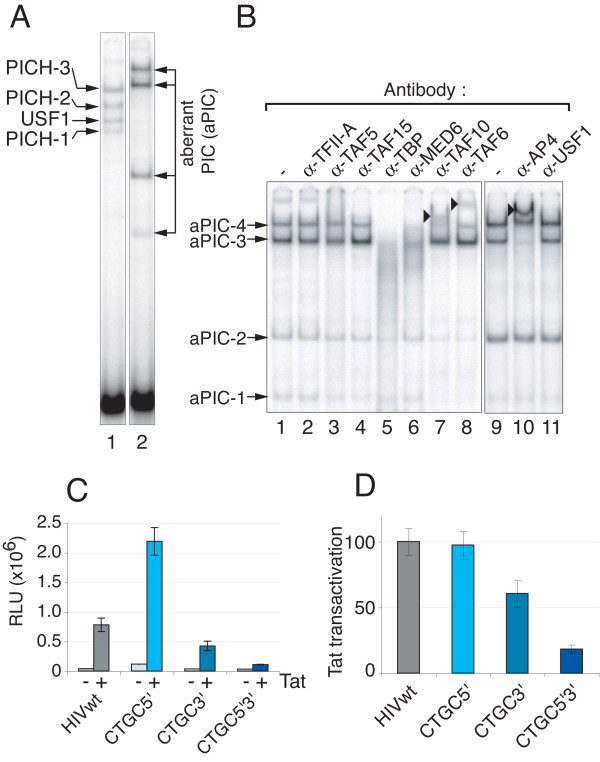
**CTGC motifs flanking the HIV TATA box are required for correct PICH formation*in vitro*and Tat*trans*-activation in living cells.** EMSAs were performed as in Figure 2. **(A)** EMSA mobility comparison of complexes formed on HIVwt promoter (lane 1) and CTGC5’3’ mutant (lane 2). PICHs bound to HIVwt are indicated at the left of the panel. Complexes formed on CTGC3’5’ mutant are indicated at the right as aberrant pre-initiation complexes (aPIC) **(B)** For supershift assay with the CTGC5’3’ radiolabelled probe, no antibody (lanes 1 and 9) or the indicated antibodies (lanes 2–8 and 10–11) were added in the reaction. Arrowheads indicate supershifted bands. **(C)** Basal and Tat transactivated promoter activity and **(D)** Tat transactivation are measured by luciferase activity in extracts of HeLa cells as in Figure 6C and D. Mutations in the CTGC motifs are shown in shades of blue.

Since the above mutational analysis showed that neither USF-1 binding nor an intact E-box was required for Tat-responsive transcription, we next asked what *cis*-acting DNA sequences within TASHET are essential to respond to Tat. Building upon previous mutational studies [18-20,45], in addition to natural TASHET conservation (Figure 1C), we have performed an extensive mutational analysis of TASHET sequences flanking the HIV TATA box (unpublished results). For simplicity, here, we present a selective subset of the most informative mutations focusing on the three CTGC motifs that flank the HIV TATA box (Figure 7A). Since previously reported individual point mutations within these motifs showed no effect on Tat *trans*-activation [45], we postulated that mutations to more than one CTGC motif may be required to reveal their impact. We mutated the cytosine at position one and the guanine at position three of the CTGC motifs (Figure 7A) as these are highly conserved in natural HIV isolates (Figure 1C). To test the impact of mutating the conserved CTGC motifs on PICH formation in EMSA, we challenged PICH bound to wild type TASHET with unlabelled competitors bearing these point mutations within the CTGC motifs (Figure 7B and 7C; see also Additional file 4A for numeric quantification). Mutation of the 5’ CTGC motif only slightly decreased affinity of PICHs for TASHET since it competed nearly as well as wt competitor for PICH (Figure 7B lane 2 versus lane 5). Mutation of the two 3’ CTGC motifs decreased the affinity of PICH-2 and PICH-3 and USF-1 for TASHET, leading to a weaker competition (Figure 7B, lane 3). Finally, the combination of mutation in the 5’ and the two 3’ CTGC motifs resulted in a strong reduction in the affinity of PICHs for TASHET (Figure 7B, lane 4). We next radiolabelled TASHET DNA bearing point mutations in all three CTGC motifs for use in EMSA with HeLa cell nuclear extracts. The mobility and intensity of the resulting complexes were remarkably distinct from those forming on the wild type TASHET (Figure 8A, lane 2 versus lane 1). We refer to these aberrant complexes collectively as aPIC (Figure 8A and 8B) to indicate that they do not allow Tat-responsive transcription (see below). To probe the constituents of these altered aPIC, we again used supershift analysis. We found no complexes to be affected by TFIIA antibodies (Figure 8B, lane 2). Importantly, these aberrant complexes were inhibited by the addition of antibodies raised against classical PIC subunits like TBP and MED6 (Figure 8B, lane 5 and lane 6; see also Additional file 4B for quantification). The lowest mobility complex was also supershifted by antibodies against TFIID subunits TAF10 and TAF6 (Figure 8B, lane 7 and lane 8). The formation of the major aPIC complex was slightly but reproducibly, reduced by the addition of antibodies against TAF5 (Figure 8B, lane 3) and TAF10 (Figure 8B, lane 7). The aPIC formed on TASHET bearing mutated CTGC motifs contained no USF-1 (Figure 8B, lane 11). This result is expected since the E-box embedded within the 3’ CTGC motifs is destroyed by these point mutations (Figure 7A and Figure 6A). In contrast AP4 antibodies resulted in a strong supershift of the major complex binding TASHET with mutated CTGC motifs (Figure 8B, lane 10 versus lane 9). Together, these observations indicate that the CTGC motifs flanking the HIV TATA box are required for correct PICH formation. Furthermore, the mutation of these flanking CTGC motifs results in aberrant complexes that retain at least some of the classical PIC components plus the transcription factor AP4.

To test the effects of the mutations that altered PICH formation *in vitro* on Tat *trans*-activation in living cells, we again employed co-transfection of reporter constructs carrying the full length HIV LTR driving luciferase with a Tat expression vector in HeLa cells. Co-transfection of a construct bearing point mutations within the single 5’ CTGC increased basal HIV LTR-directed transcription (Figure 8C, mutation CTGC5’) without significantly increasing Tat *trans*-activation (Figure 8D). This result mirrors the previously reported effects of mutations immediately 5' of the TATA box [46]. Mutation of the two 3’ CTGC motifs resulted in a significant drop in Tat *trans*-activation (Figure 8D, mutation CTGC3’) without significantly changing basal transcription (Figure 8C). A construct bearing mutations in all three flanking CTGC motifs resulted in highly impaired Tat *trans*-activation (Figure 8D, mutation CTGC5’3’), yet only slightly decreased basal HIV LTR-driven transcription (Figure 8C). To rule out indirect effects on Tat *trans*-activation by grossly altered transcriptional start sites, the start sites of transcription for mutated core promoters were analyzed by primer extension in transfected HeLa cells. The major start site of transcription was found to be unchanged with all mutations used (Additional file 5). We concluded that the CTGC DNA motifs flanking the TATA box are essential for Tat *trans*-activation of the HIV promoter.

### CTGC DNA motifs within TASHET are essential for HIV gene expression in single-round infection assays

**Figure 9:**
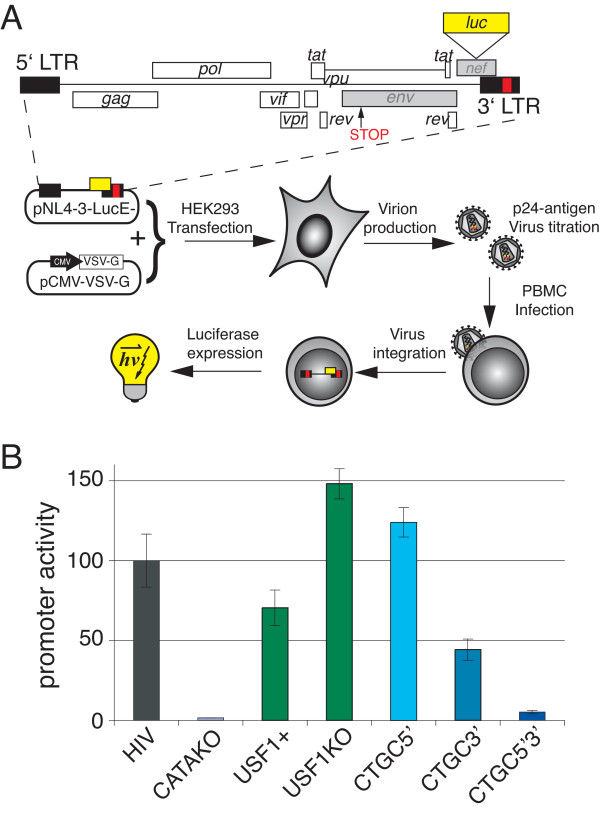
**The CTGC motifs of TASHET are essential for activated HIV transcription in single-round infection assays. (A)** Mutations have been introduced into the 3’LTR of pNL4-3-LucE- (in red). Viral proteins whose expression is deficient in this construct are shown in grey (*env*, *nef*). The mutated pNL4-3-LucE- constructs were co-transfected together with a plasmid expressing VSV-G envelope into HEK293 packaging cells for virion production. Culture supernatant was titrated for viral content by ELISA p24-antigen titration to normalize the quantity of virus for PBMC infection. Activated PBMCs were infected, allowing genomic integration of the virus. Luciferase expression corresponding to retroviral promoter activity was measured 48 h later. **(B)** RLU were measured and normalized to proviral content as measured by qPCR of an LTR sequence in infected cells extract. Results were expressed as a percentage of wt HIV luciferase activity.

To further test the importance of the CTGC motifs flanking the HIV TATA box in a physiologically relevant context, we next employed previously described single round infection assays [47,48]. We used pseudotyped viral particles to infect PBMC from healthy donors as shown schematically in Figure 9A. This system relies on a luciferase reporter gene that replaces the HIV *nef* gene with the VSV-G envelope protein provided by co-transfection of packaging cells. The advantages of this system are three fold. Firstly, primary PBMC are used and represent the major physiological targets for natural HIV infection. Secondly, the HIV promoter is integrated in a single copy into the chromatin context as in a natural infection. Thirdly, Tat expression is driven by the proviral HIV promoter and is therefore supplied in an amplification loop identical to a natural infection.

To define the impact of TASHET point mutations on proviral HIV transcription in PBMCs, we isolated the cells and activated them with mitogens IL-2 and PHA before infection with viral particles bearing point mutations in the 3’ LTR that are subsequently copied into the 5’ LTR by HIV reverse transcriptase before integration into the host cell genome (Figure 9A). Luciferase activity was measured to monitor HIV gene expression forty-eight hours post infection. An HIV genome with a mutated TATA box served as a negative control and resulted in very low levels of luciferase activity (Figure 9B, CATAKO). Point mutations that increase USF-1 binding (Figure 6) decreased HIV expression to approximately 70% of wild type levels (Figure 9B, USF+). Point mutations that prevent USF-1 binding but retain PICH-2 binding (Figure 6) resulted in Tat-activated HIV gene expression that was measurably higher than that of wild type promoter (Figure 9B, USF1KO). We conclude that in the single round infection assay in PBMC USF-1 binding is not essential for activated HIV gene expression. Mutation of the 5’ CTGC motif alone had little effect on HIV gene expression and even slightly increased luciferase activity in infected PBMC (Figure 9B, CTGC5’). Mutations within the two 3’ CTGC motifs reduced HIV gene expression by approximately one half (Figure 9B, CTGC3’). Importantly, the point mutation of the three flanking CTGC motifs reduced HIV expression levels strongly showing that, in the proviral chromatin context and in primary PBMC, these motifs are essential for activated HIV gene expression.

### TAR RNA prevents stable PICH-2 binding to TASHET DNA

**Figure 10:**
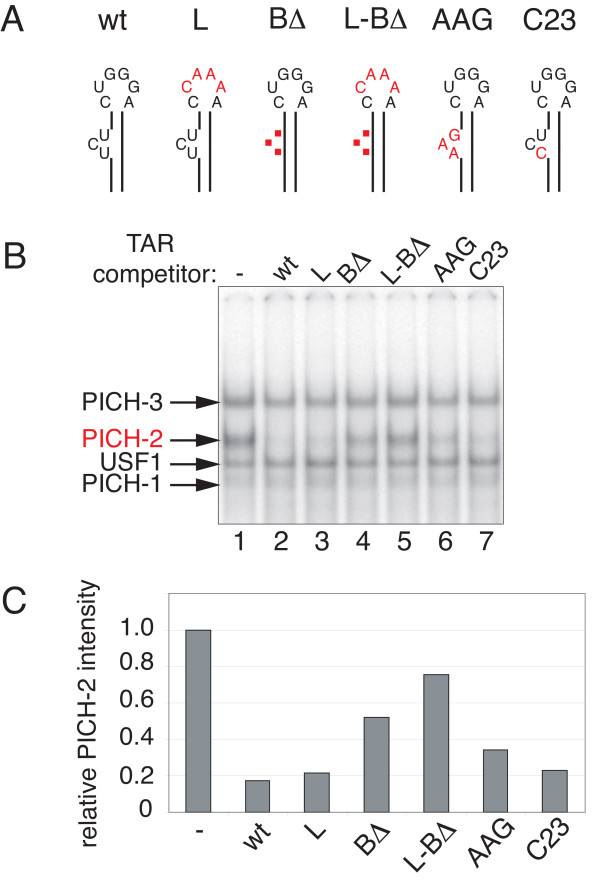
**TAR RNA prevents stable PICH-2 binding to TASHET DNA. (A)** The stem loop structure of TAR RNA mutants is depicted (nt 1 to 83) with the name of the mutation above. Mutations in the loop and/or in the bulge are shown in red. The red dots stand for nucleotides deletions. **(B)** 40 fold molar excess of indicated TAR mutant *in vitro* transcripts have been added in EMSA reactions that were performed as detailed in Figure 2. **(C)** PICH-2 intensity has been quantified by band density measurement on EMSA autoradiography. Results are expressed relative to PICH-2 intensity in the control lane 1 containing no TAR that was set to one (−).

Given that the CTGC motifs of TASHET are required for Tat responsive transcription, and recent observations that TAR RNA can displace complexes formed on the HIV promoter [11], we next asked whether TAR has an impact on the formation of the specialized PIC (PICH) that recognize TASHET. TAR RNA and mutations thereof (Figure 10A) were transcribed *in vitro*, purified and then added to EMSA with radiolabelled TASHET DNA. Wild type TAR RNA resulted in a loss of binding of PICH-2 (Figure 10B, lane 2 versus lane 1). To determine the sequences of TAR required to prevent PICH-2’s stable binding to TASHET, we employed mutations in the loop and bulge region of TAR previously shown to prevent Tat *trans*-activation [49-51]. TAR RNA with mutated loop sequence, like the wild type, was able to prevent stable PICH-2 – TASHET interaction (Figure 10B, lane 3 versus lane 2). In contrast TAR RNA lacking the three nucleotide bulge known to bind Tat was significantly less able to prevent PICH-2 – TASHET interaction (Figure 10B, lane 4 versus lane 2). Mutation of the bulge and loop sequences together nearly abolished TAR’s impact on stable PICH-2 formation (Figure 10B, lane 5). Changing the nucleotide sequence of the bulge from UCU to AAG only slightly reduced the ability of TAR to destabilize the PICH-2 – TASHET interaction (Figure 10, lane 6). We concluded that the structure of the bulge of TAR is more critical than its nucleotide sequence for the destabilization of PICH-2 – TASHET binding. This conclusion is further supported by the single nucleotide change at cytosine 23 that did not alter TAR’s impact on PICH-2 – TASHET interactions (Figure 10B, lane 7). The impact of TAR on PICH-2 – TASHET interaction was quantified and is shown in Figure 10C. To test the specificity of TARs on the PICH-2 complex, we also tested their impact on the interaction of canonical PIC with the AdMLP promoter and found no effect on any complexes (Additional file 6). Furthermore, the complexes binding to the CTGC5’3’ TASHET mutation that no longer responded to Tat were not affected by the presence of TAR RNA (Additional file 6). TAR RNA has recently been shown to displace a 7SK snRNP from the HIV promoter [11] raising the possibility that PICHs at the HIV core promoter could contain 7SK RNA. To test this possibility we used RNase H-directed degradation of 7SK in HeLa cell nuclear extracts. Efficient 7SK degradation had no significant effect on PICH formation in EMSA, showing that 7SK is not an integral component of PICHs (Additional file 7). Together these results show that TAR RNA, via its bulge sequence, can prevent stable binding of PICH-2 to TASHET DNA.

## Discussion

The data we present here show for the first time that the TATA box of HIV and adjacent sequences of HIV essential for Tat trans activation (TASHET) is recognized by cellular pre-initiation complexes (PICHs) that: 1) are distinct from canonical PIC that recognize the model AdMLP, 2) require the flanking CTGC motifs for their accurate formation, and 3) include PICH-2 whose stable binding to TASHET is disrupted by HIV TAR RNA. TASHET has been shown to play a pivotal role in governing Tat-activated HIV transcription in cell culture [16-21,37] and by logical extension is thought to impact the dynamics of latency *in vivo*[52]. Moreover, previous studies have provided a proof-of-principle that DNA sequences within the TASHET element of the HIV core promoter can be specifically targeted by polyamides to modulate HIV transcription [29,53,54]. The host cell PICH complexes identified here are of importance as an essential step in the activation of HIV transcription, and also as a source of potential new drug targets for the eradication of HIV from infected individuals.

The functional diversity of core promoters and of the transcription factors that they bind is increasingly recognized as an important contributor to genomic regulation [6,30-35]. Of the known consensus core promoter elements (DPE, DCE, BRE, XCPE1, etc.), the HIV promoter contains only an identified TATA box [25] and a non-classical initiator (Inr) element [55]. In contrast to TASHET [16-21], the HIV Inr can be replaced by a heterologous AdMLP Inr for Tat-responsive transcription [56]. The data presented here excluded an essential role for USF-1 binding to the 3' E box in Tat *trans*-activation (Figure 6). Two other CTGC motifs were found in upstream HIV LTR motifs termed RBEIV and RBEIII elements [57], but were not required for Tat *trans*-activation [58]. The 3’ CANNTG E-box is embedded within the 3’ palindromic GCAGCTGC motif (Figure 6A). The 3’ E-box displays relatively low sequence conservation [22,39] (Figure 1C). Our data could help explain why a GCAGC**C**GC variant is frequently found in natural HIV isolates (Figure 1C), yet weakens the consensus E-box GCAGCTGC, if positive selection pressure to maintain PICH-2 - CTGC contacts can prevail over selection to maintain USF-1/AP4 - E-box interactions in HIV infected individuals.

We postulate two mechanisms to account for the specific functional and DNA binding properties displayed by the PICH described herein. First, the known Pol II general transcription factors (GTFs) could have unique affinities or conformations when bound to TASHET that confer functional specificity. There are precedents for core promoter specific GTF function, for example TFIIA, that we have found as a component of PICH-2, has been shown to have positive or negative effects on transcription depending on the core promoter sequence [59]. A second alternative is that unknown accessory (non-GTF) host cell proteins could bind to TASHET to confer Tat-responsiveness. The two possible mechanisms are not mutually exclusive and could contribute together to PICH specificity. Certain TBP-associated factors (TAFs) can recognize core promoter elements [32,60]. Our supershift analysis suggests that stable PICH formation on TASHET requires at least some core TAFs. The PICH appear to bind less tightly to TASHET compared to canonical PIC to the AdMLP, since antibodies to TFIID subunits disrupt PICH – TASHET interactions more readily than PIC – AdMLP interactions in EMSA (Additional file 1). These results are compatible with chromatin immunoprecipitation (ChIP) results obtained from Tat-expressing cells showing that TAF occupancy of the HIV core promoter is lower than that of TBP when compared to an adenovirus E1b core promoter [61]. Nevertheless, both TFIID [62] and TAFs [63] have been shown to bind the HIV core promoter *in vitro*. The ratios of TBP/TFIID occupancy measured by ChIP must be interpreted in light of the fact that association of TBP with TFIID is highly dynamic both *in vitro*[64] and in living cells on model promoters [65].

Cellular complexes have been previously reported to bind TASHET [37,57,66], but their identity has remained enigmatic due to their enormous size, subunit complexity, and their dynamic nature on core promoters. The minimal set of classical GTFs required for PIC formation and transcription is considered to be 70 polypeptides [67], and proteomic studies of yeast PIC composition imply that hundreds of proteins are involved [68]. TASHET’s key role in HIV transcription was discovered more than two decades ago [16], yet the complexes recognizing it have remained out of reach. Based on the GTFs they contain, PICH-1 (e.g. TBP, TAFs), PICH-2 (TBP, TAFs, TFIIA) and PICH-3 (TBP, TAFs, Pol II) likely correspond to intermediates in the PIC assembly pathway [69]. The availability of a tractable EMSA to detect PICH, the demonstration that PICH contain classical GTFs yet are distinct from canonical PIC, together with the definition of the nucleotides essential for their formation, opens the door for molecular genetic, biochemical and proteomic dissection of PICH composition.

**Figure 11:**
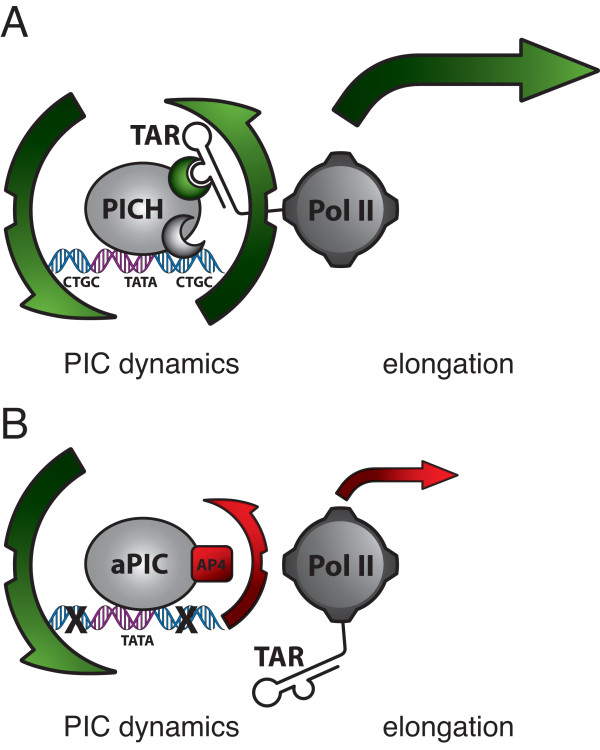
**A hypothetic model for PICH function in Tat*trans*-activation. (A)** A highly simplified schema illustrating the coupling of pre-initiation complex (PIC) dynamics with transcriptional elongation. On the wild type HIV core promoter nascent TAR RNA contacts PICH to increase the rate of dissociation of PICH-2 (upward green arrow), thereby increasing the rates of the subsequent cycle of PICH formation and potentially Pol II escape. **(B)** In contrast, on core promoters lacking functional CTGC motifs, aberrant complexes (aPIC), containing AP4, are slow to dissociate (upward red arrow) and TAR has no impact on them (see text for details).

By revealing that PICH formed on the HIV core promoter are distinct in electrophoretic mobility and DNA binding specificity from PIC that bind the prototypical AdMLP, our data provide a mechanistic explanation for the specific requirement of TASHET in Tat *trans*-activation. Physical interactions between HIV Tat and Pol II PIC components including TBP [27,28] and TFIIB [70,71] have been previously reported. Furthermore, recombinant Tat was shown to enhance the sarkosyl-resistance of purified TFIID – TFIIA complexes on HIV TATA box region in EMSA *in vitro*[26]. To date we have observed no significant effect of recombinant Tat on endogenous PICH behaviour in EMSA. One reason that could explain a lack of interaction between Tat and the PICHs is that the TASHET DNA employed in our EMSAs lacks the SP1 sites that are essential for recruitment of 7SK snRNP [11] that can interact with Tat [72]. Tat’s capacity to enhance transcription elongation rates via P-TEFb is well established [15], however in several experimental settings Tat can also positively influence transcription initiation [73]. Our analysis did not distinguish between the relative contributions of enhanced elongation versus enhanced initiation. Instead, the demonstration that TAR RNA can influence PICH-2 binding to TASHET reinforces and extends recently proposed models [11,74] for Tat function in which the initiation and elongation steps of transcription are mechanistically coupled (Figure 11A).

Our data show that TAR RNA, via its bulge structure, can prevent stable association of PICH-2 with TASHET in EMSA (Figure 10). In principle TAR could act by blocking association of PICH-2 with TASHET or by accelerating the dissociation of PICH-2 from TASHET. We favour a role for TAR in the dissociation from TASHET because PICH-2 binding correlates positively with Tat-responsiveness (Figure 6, and data not shown), and because TAR decoys do not inhibit PIC formation *in vitro*[75]. We propose a dynamic model in which cellular PICH specifically recognize TASHET via its CTGC motifs. Nascent TAR transcribed by Pol II facilitates the departure of PICH-2, in turn accelerating the next cycle of PIC formation (Figure 11B). This model is compatible with the model recently proposed by D’Orso *et al.* including the expulsion of 7SK snRNP from the HIV promoter by TAR [11]. A dynamic model is also compatible with the observation that point mutations to the CTGC motifs result in aberrant but stable aPIC formation and a failure to respond to Tat (Figure 8). The presence of AP4 in aPIC was unexpected since the 3’ E-box is destroyed by these mutations. A plausible explanation is that AP4 may be recruited into non-productive PICH via protein-protein interactions. The dynamic model we propose for the impact of both PICH association and disassociation on Tat *trans*-activation is consistent with previous genetic observations that appropriate PIC destabilization is necessary for activated transcription in yeast [76] and recent reports showing that transcription factor dynamics are important in transcriptional regulation [77,78].

Our results shed new light on the mechanisms that control HIV gene expression, but also have broader implications for the combinatorial control of cellular gene expression. Biologically, the sequence of core promoters has been shown to dictate: 1) the differential response to activators [79], 2) the response to cell-type specific enhancers [80], and 3) the alternative splicing of the transcribed pre-mRNA [81]. The composition of core promoter-binding complexes is increasingly recognized as being very heterogeneous [31-33,82]. In addition, the range of core promoter *cis*-acting DNA sequences is also known to be highly complex [6,60]. To our knowledge, to date no biochemical data had been reported showing that distinct core promoters bind PIC that are functionally distinct, yet contain common PIC components such as TBP and Pol II. The data presented here link a specific biological outcome (Tat *trans*-activation), to a core promoter sequence (the CTGC motifs) and also to a specialized core promoter-binding complex (PICH-2). The broader mechanistic implication is that core promoter complexes, despite the fact that they contain common classical GTFs, can confer very distinct transcriptional responses.

## Conclusions

The regulation of HIV transcription dictates viral latency versus active replication. The viral Tat *trans*-activating protein is essential for activated HIV transcription. A long-standing unanswered question concerning the mechanism of Tat *trans*-activation is: why can the HIV core promoter TATA box region (TASHET) not be functionally replaced by heterologous TATA elements? We report here that CTGC DNA motifs in the HIV core promoter are essential for the formation of specialized Tat-responsive pre-initiation complexes (PICH). PICH-2 contains the general transcription factor TFIIA and its stable association with TASHET is prevented by the presence of HIV TAR RNA. The detection of Tat-responsive PICH binding complexes provides an essential step forward towards the full elucidation of the mechanisms underlying activated HIV transcription, and paves the way for the identification of new molecular targets for therapies to eradicate latent HIV.

## Methods

### Antibodies

Antibodies used in this study were raised against: AP-4 (sc-18593), Med6 (sc-9434), TFIIA-γ (sc-5316), USF-1 (sc-229), all from Santa Cruz, CA; Pol II (8WG16) from Covance (Emeryville, CA); SMARCA3 (BL825) from Bethyl Laboratories (Montgomery, TX); TFII-I (#4562) from Cell Signaling (Beverly, MA). Monoclonal antibodies against TFIID subunits were generous gifts from Dr. Laszlo Tora and have been described [83-85] : TBP : TBP-1 (2 C1), TBP-2 (4 C2), TBP-3 (3 G3) ; TAF4 (20TA) ; TAF5 (1TA) ; TAF6 (25TA) ; TAF10 (2B11) ; TAF15 (2B10).

### Plasmids

pCMV-Tat has been described [86]. To obtain the negative control lacking Tat coding sequence, pCMV-Tat was linearized with XhoI and SalI restriction enzymes and blunted with the Klenow enzyme before religation. pHIV-RL is based on the pHRL-null vector (Promega #E6231) whose XbaI and SphI sites were eliminated by restriction enzymes, Klenow digestion and religation. The HIV-1 LTR of pU3S [57], derived from pHIVSCAT [18], was cloned into the XhoI and HindIII sites of the previously modified pHRL-null. The resulting plasmid was digested with XhoI and KpnI and treated with Klenow enzyme before religation to eliminate the 5' XbaI site of the LTR. All the Renilla luciferase mutant constructs were based on pHIV-RL, by replacing the wild-type core promoter sequence between the XbaI and SphI sites with 35 bp synthetic oligonucleotides bearing the various mutations. Key mutations have been cloned in pNL4-3-LucE- (kindly provided by Michel J. Tremblay, Université Laval, Québec) for pseudotyped virus production. To facilitate the mutation, the XhoI-NcoI portion of pNL4-3-LucE- has been cloned into phRLnull and directed mutagenesis was performed by PCR as previously described [87]. The XhoI-NcoI portion was then reintroduced into pNL4-3-LucE- by standard subcloning. The envelope encoding plasmid pCMV-VSV-G was also a gift of Dr. M.J. Tremblay. Mutated TAR RNAs based on previously reported mutations [49-51] were cloned into the SphI and SacI sites of the pHIV-RL backbone using synthetic oligonucleotides bearing mutations. In the ΔTAR mutant, 30 nucleotides were deleted in TAR sequence by SacI-HindIII digestion, Klenow blunting and religation.

### Cell culture and nuclear extracts

HeLa cells (ATCC # CCL-2) were grown in DMEM supplemented with 2.5% FBS and 2.5% NBCS (Wisent). HEK293 cells (ATCC #CRL-1573) were grown in DMEM containing 10% FBS. Peripheral blood mononuclear cells (PBMC) were isolated from healthy donors by lymphocyte separation medium (Wisent) according to manufacturer’s instructions and stimulated 3 days with PHA-L (1 μg/ml, L2769 Sigma) and IL-2 (30 U/ml, Sigma or F081 Bioshop) before viral infection or nuclear extracts preparation. Nuclei were prepared from activated PBMC [88] and HeLa cells [89] as previously described. PBMC and HeLa cell nuclei were then used to prepare nuclear extracts according to the protocol of Dignam *et al*. [89].

### In vitro transcription

To generate TAR transcripts, the sequence was first amplified by PCR using a sense primer containing a T3-polymerase recognition site. Three 50 μl reactions were prepared, each containing 200 ng of matrix plasmid, 1 mM of each dNTPs, 400 nM of sense and reverse primer, 2.5 U of Pfu turbo enzyme in 1x reaction buffer (NEB, Ipswich MA). PCR products were pooled and purified by phenol-chloroform extraction and ethanol precipitation. The obtained template was reverse transcribed for 1 to 2 h at 37 °C in a mix containing 0.5 mM of each rATP, rCTP and rUTP, 0.1 mM rGTP, 0.2 mM Cap analog, 12.5 pmol (10 μCi) of α-^32^P UTP, 2 μl RNA guard, 34U of T3 RNA-polymerase in 100 μl of 1X transcription buffer (40 mM Tris–HCl pH7.9, 6 mM MgCl_2_, 2 mM spermidine, 10 mM DTT). The transcription product was mixed with formamide dye (1 mg/ml of each bromophenol blue and xylene cyanole, 10 mM EDTA in deionized formamide), boiled for 90 seconds and immediately chilled on ice, then loaded on a pre-equilibrated denaturing 8 M urea- 4.75% polyacrylamide gel in TBE 1X and run for 45 min at 300 V. Full length TAR transcripts were identified by autoradiography and then excised from the gel for extraction with 300 μl crunch solution (300 mM Na Acetate, 0.2% SDS) on a rocking table twice for 20 min. Pooled supernatants were phenol-chloroform extracted twice, ethanol precipitated, resuspended in water and the radioactive counts were measured for the calculation of RNA concentration.

### Electrophoretic mobility shift assay (EMSA)

The specific EMSA protocol used in this study has been described in detail [36]. Briefly, 20 μg nuclear extracts were mixed together with 4μg of acetylated BSA (Promega #R3961), 2 μg of Poly(dI-dC)·Poly(dI-dC) (Sigma) and 1.8pmol of nonspecific double-stranded oligonucleotide (sense : GATCCGGAGTACTTCAAGAACG; reverse : GATCCGTTCTTGAAGTACTCCG) in a final volume of 20 μl of binding buffer (20 mM Hepes, 5 mM MgCl_2_, 8% glycerol, 100 mM KCl), for 5 minutes on ice. For competition assays, TAR RNA or 200pmol (unless otherwise specified) of unlabelled competitor oligonucleotide were added to the reaction. 1pmol of labelled doubled-stranded promoter (35 bp) were added to reaction for 15 minutes at room temperature. For supershift assays, 1 to 4 μl of specific antibody were added to the sample, and the reaction was continued on ice for 1 additional hour. Samples were then loaded on a native 4.5% polyacrylamide gel and complexes separated for 3.5 hours at 150 Volts. For RNase H directed degradation of 7SK RNA within the nuclear extracts used in EMSA, nuclear extracts were first incubated with anti-sense oligonucleotide and 10 U of RNase H (NEB) for 1 h at 30°C in binding buffer. The remaining constituents were then added as indicated above (BSA, Poly(dI-dC)·Poly(dI-dC), non specific oligonucleotide and finally radiolabelled oligonucleotide) and the reaction was allowed to continue as described above. The anti-sense oligonucleotides for 7SK RNase H directed digestion have been described [90].

### RT-PCR on EMSA samples

To check for proper and specific RNA degradation, the EMSA reaction was doubled, and RNAs were recovered from one half of the resulting volume by trizol extraction. The samples were DNase I treated (Promega) for 30 min at 37°C. RNAs were reverse transcribed by MMuLV-RT (Roche) using random hexamer-primers according to manufacturer’s recommendations. PCR was performed on 1/10 of the obtained cDNA to amplify 7SK-snRNA : forward GGATGTGAGGCGATCTGGC ; reverse : AAAAGAAAGGCAGACTGCCAC; or U6snRNA : forward CTCGCTTCGGCAGCACATATAC ; reverse GGAACGCTTCACGAATTTGCGTG.

### Transfection and luciferase assay

For Renilla Luciferase reporter assays, HeLa cells were transfected in 96 well plates with DMRIE-C (Invitrogen,) according to the manufacturer’s recommendations. 12000 cells were seeded into 96 well plates the day before transfection in complete medium. 50 ng of pHIV-RL construct, together with 15 ng of either pCMV-Tat or the empty control vector were transfected, complexed with 0.2 μl of DMRIE-C transfection reagent in a final volume of 100 μl of OptiMEM (Invitrogen). Two days later, the Renilla Luciferase assay system (# E2810, Promega, Madison, WI) was used according to the manufacturer’s instructions to lyse and measure luminescence on an automated luminometer (BMG Labtech, Ortenberg, Germany).

### Primer extension

HeLa cells were transfected in 6 well plates with DMRIE-C (Invitrogen,) according to the manufacturer’s recommendations. 5 X 10^5^ cells were seeded per well the day before transfection in complete medium. 1.25 μg of pHIV-RL construct, together with 250 ng of either pCMV-Tat or the empty control vector were transfected, complexed with 4 μl of DMRIE-C transfection reagent in a final volume of 1.5 ml OptiMEM (Invitrogen). RNAs were extracted with Trizol (Invitrogen) reagent after 24 h. 5 μg of total RNA was used in primer extension reactions for 1 h at 42 °C in a buffer containing 1.25 mM Tris pH 8.0, 1.75 mM KCl, 5 mM DTT, 10 mM MgCl2, 125 μM of each dNTP, 25 μg/ml Actinomycin D, 5U of AMV Reverse-Transcriptase (Roche) and 20 ng of a PNK radiolabelled reverse primer specific for TAR (5’ GCTTTATTGAGGCTTAAGCAGTG3’). The ethanol precipitated pellet was resuspended in formamide dye, boiled and loaded on an 8 M urea 9% polyacrylamide denaturing sequencing gel.

### Virus production

The day before transfection, 4 X 10^6^ HEK-293 packaging cells were seeded on 100 mm dishes in 9 ml complete medium. Calcium phosphate transfection was performed by mixing together 1.5 μg pVSV-G and 13.5 μg pNL4-3-LucE- based constructs in 500 μl of 250 mM CaCl_2_ and 500 μl HBS 2X (280 mM NaCl, 50 mM Hepes, 1.5 mM Na_2_HPO_4_, pH7.08). The precipitates were allowed to form for 2 minutes and immediately added drop-wise on top of cell culture medium. Two days later, the virion containing medium was recovered and filtered through a 0.45 μm filter and kept frozen at −80°C. Virion content was estimated using an in house enzyme-linked immunosorbent assay (ELISA) for the viral major core protein p24 that has been previously described [47].

### Viral reporter assays

PBMC were activated for 3 days before infection. 10^6^ cells were directly resuspended in the minimal volume of complete medium and virus stock to add 100 ng of p24 per 10^6^ cells. Pre-infection was allowed to occur at 37°C for 1 hour. Complete medium was added to reach 10^6^ cells/ml. The infected cells were further incubated for the desired time. Cells were centrifuged, washed twice with PBS; 1/5 were used for qPCR, and 4/5 for luciferase assay. The infected cells were resuspended in 100 μl 1X Lysis buffer (25 mM Tris, 2 mM DTT, 1% Triton X-100, 10% glycerol) and luciferase activity was measured as described above. 1/5 of the infected cells were lysed by resuspension of cell pellets in 200 μl of qPCR lysis buffer containing Tris–HCl, pH 8, 10 mM, and Polyoxyethylen 10 Lauryl-ether 0.1% (Sigma, P-9769) [91]. Proteinase K was added to a final concentration of 100 μg/ml and samples were incubated at 65°C for 2 h followed by 15 min at 95°C. The obtained extracts were directly used for qPCR of HIV promoter for quantification of proviral copy number (sense : 5’-CTGCTGACATCGAGCTTTCTACAAGGG-3; reverse: 5’-AGGCTCAGATCTGGTCTAACCAGAGAG-3’). To normalize for proviral content, standard samples of known pNL4-3-LucE- copy number were included in each qPCR run. 2 μl of extracts were used as template for qPCR reaction, in a 20 μl reaction containing 150 nM of each primer, 200 μM of each dNTP and 2U of KlenTaq in reaction buffer (6 mM Tris–HCl pH 8.3, 25 mM KCl, 4 mM MgCl_2_, 75 mM D + Trehalose dihydrate (Bioshop Canada), 0.1% Tween 20. 0.1 mg/ml BSA ,0,1X Sybr Green (Invitrogen, Eugene OR)). Primer sequences for mutated HIV promoter constructs are provided in Table 1. Experiments with human cells were conducted in accordance to the Helsinki Declaration, with prior written consent from donors, and as approved by the Ethics Committee of the Université de Sherbrooke.

**Table 1:** Oligonucleotide sequences for EMSA and pHIV-RL constructs

**Name**	**Sequence**
HIV wt	CTAGATGCTGCATATAAGCAGCTGCTTTTTGCATG
TATAKO	CTAGATGCTG**AGAGCTC**GCAGCTGCTTTTTGCATG
USF-1+	CTAGATGCTGCATATAAGCA**CG**TGCTTTTTGCATG
USF-KO	CTAGATGCTGCATATAAGC**G**GC**C**GCTTTTTGCATG
AdMLP	CTAGA**GGGGCTATAAAAGGGGGTGGGGGCG**GCATG
HSP70	CTAGA**CGACTTATAAAAGCCCAGGGGCAAG**GCATG
CTGC-5’	CTAGATG**A**T**C**CATATAAGCAGCTGCTTTTTGCATG
CTGC-3’	CTAGATGCTGCATATAAG**G**A**TA**T**C**CTTTTTGCATG
CTGC-5’3’	CTAGATG**A**T**C**CATATAAG**G**A**TA**T**C**CTTTTTGCATG

Primers were designed to create a double-stranded template for radioactive labelling for use in EMSA or for direct cloning into XbaI and SphI sites. For brevity only the positive strand oligos are shown. Bold face type indicates nucleotides mutated from the wild type sequence.

## Abbreviations

AdMLP, Adenovirus major late promoter; aPIC, Aberrant pre-initiation complex (of RNA polymerase II); EMSA, Electrophoretic mobility shift assay; PBMC, Peripheral blood mononuclear cells; PIC, Pre-initiation complex (of RNA polymerase II); PICH, Pre-initiation complex of HIV; Pol II, RNA polymerase II; RLU, Relative light units; snRNP, Small nuclear ribonucleoprotein complex; TAF, TBP-associated factor; TBP, TATA binding protein; TASHET, TATA box of HIV and Adjacent Sequences of HIV Essential for Tat trans-activation; Wt, Wild type.

## Competing interests

The authors declare that they have no competing interests.

## Author’s contributions

EW performed the majority of experiments, supervised other experimenters and wrote the manuscript. MCD performed molecular cloning, viral infection assays and their analysis. IN performed EMSA, mutagenesis and luciferase assays. AM performed quantification of EMSA data. ND supervised the viral infection assays and provided expertise and reagents. BB performed EMSA, designed the experiments, supervised the research and wrote the manuscript. All authors read and approved the final paper.

## Supplementary Material

Additional file 1 **Figure S1.** Quantification of complex intensity in EMSA for Figures 4 and 5**.** Band intensity or entire lane intensity was quantified by phosphorimager analysis of EMSA. (A) A table showing the percentage of signal reduction for each PICH relative to its density in the control lane containing no antibody (0) in Figure 4. ‘-’ indicates no signal reduction. Band intensities that were reduced by one third (33%) or more relative to their controls are highlighted in orange. (B) Graphic representation of the intensity of the whole EMSA lane to show quantitative analysis of supershift signals with antibody against AP4 (left panel, blue) and TFII-A (right panel, pink) compared to the control with no antibody (black profile). Arrows and colored interspaces between curves indicate the former and new position of the supershifted band. The highest peak in the control is arbitrarily set to 100%. The peaks corresponding to PICH-3 and -2 are indicated as reference points. (C) As in A, except quantification of Figure 5 is shown (EMSA with HeLa NE). (D) As in B except quantification of Figure 5 is shown (EMSA with HeLa NE). Click here for file

Additional file 2 **Figure S2.** PICH and canonical PIC are differentially affected by antibodies directed against TFIID subunits. EMSA were performed as in Figure 2. Radiolabelled wt HIV promoter (lanes 1-4) or MLP (lanes 5-8) were used as probes. Indicated specific antibodies directed against general transcription factors were added to the EMSA reaction for supershift assays. Pre-Initiation Complexes of HIV (PICHs) are indicated at the left of the first panel. Complexes formed on AdMLP (unnamed) are indicated by arrows on the left of the second panel. Click here for file

Additional file 3 **Figure S3.** Validation of point mutations that enhance or block USF-1 binding to TASHET. EMSAs were performed as in Figure 2. (A) Radiolabelled wt HIV promoter was incubated with HeLa nuclear extracts with or without addition of increasing amounts of unlabelled competitor : 20 (lanes 2, 5, 8), 60 (lanes 3, 6, 9) and 200 (lanes 4, 7, 10) fold molar excess of the indicated competitors were added to the reaction. (B) The intensity of the PICH-2 (left panel) and USF-1 (right panel) bands have been measured on the phosphorimager analysis of EMSA from panel A and the relative amount of these two complexes have been calculated. Results are expressed starting with the intensity without competitor being set to 1. The relative complex intensity in presence of wt HIV promoter competitor is shown with a black line, as is that in the presence of USF-1+ competitor (green line) and with USF-1KO competitor (red line). The triangle on the horizontal axis stands for 20, 60 and 200 fold molar excess of competitor versus radiolabelled probe. Click here for file

Additional file 4 **Figure S4.** Quantification of complex intensity in EMSA for Figures 7 and 8. The intensity of the EMSA bands was quantified using phosphorimaging. Values in the tables express the percentage of signal reduction for each PIC relative to its density in the control lane of the EMSA. ‘-’ indicates no signal reduction. (A) Quantification of EMSA in Figure 7. PICH intensities that were reduced by one half (50%) or more relative to their controls are highlighted in red. (B) Quantification of EMSA in Figure 8. PICH intensities that were reduced by one third (33%) or more relative to their controls are highlighted in orange. Click here for file

Additional file 5 **Figure S5.** Mutations in TASHET do not affect the transcription start site position. (A) HeLa cells were co-transfected with a Tat expression plasmid and a plasmid expressing Renilla luciferase under the control of HIV wt (lane 4) or mutated promoter (lanes 5 to 11). RNAs have been extracted 24h after transfection and used in primer extension assays. Lane 2 contains only the primer, lane 3 a primer extension on untransfected HeLa RNA. Lane 1 contains the ladder whose sizes are indicated at the left. The major transcript corresponding to the expected start site is indicated by +1. (B) As in panel A, but in the absence of Tat. Click here for file

Additional file 6 **Figure S6.** TAR RNA does not affect PIC on the AdMLP or HIV with mutated CTGC motifs. EMSA were performed as in Figure 2. HIV wt promoter (lanes 1-4), AdMLP (lanes 5-8) and CTGC5’3’ (lanes 9-12) were used as probes. 20 fold molar excess of *in vitro* transcribed TAR RNA were added as competitors as indicated. Click here for file

Additional file 7 **Figure S7.** 7SK snRNA is not required for PICH binding to TASHET. EMSA were performed as in Figure 2 except that HeLa nuclear extract was pre-incubated with increasing amounts (5, 50 and 500ng as symbolized by the triangle) of antisense oligonucleotide specific (221-241A) or non specific (221-241S) to 7SKsnRNA, after which RNase H was added to digest the RNA-DNA duplex that may have been formed. Reaction was then further performed as usual for EMSA and was divided in two when ready for electrophoresis. (A) EMSA followed by autoradiography on one half of the reaction and (B) qPCR of 7SK snRNA and U6 snRNA as a control on the other half. Click here for file
